# Two new species of the subgenus *Trichodolichopeza* Alexander, 1917 from China, with a key to Asian species (Diptera, Tipulidae, *Dolichopeza*)

**DOI:** 10.3897/zookeys.1285.177963

**Published:** 2026-07-17

**Authors:** Qifei Liu, Junling Geng

**Affiliations:** 1 Biological Control Research Institute, Fujian Agriculture and Forestry University, China Fruit Fly Research and Control Center of FAO/IAEA, State Key Laboratory of Agricultural and Forestry Biosecurity, Fuzhou,350002, China Biological Control Research Institute, Fujian Agriculture and Forestry University, China Fruit Fly Research and Control Center of FAO/IAEA, State Key Laboratory of Agricultural and Forestry Biosecurity Fuzhou China; 2 Fujian Provincial Environmental Monitoring Central Station, Fuzhou 350003, China Fujian Provincial Environmental Monitoring Central Station Fuzhou China

**Keywords:** Dolichopozinae, hypopygium, new combination, new species, Tipulinae

## Abstract

Two new species of the subgenus Dolichopeza (Trichodolichopeza) from China are described and illustrated: D. (T.) caudata**sp. nov**. and D. (T.) uncinata**sp. nov**. Dolichopeza (Nesopeza) fabella Alexander, 1937 is transferred to the subgenus *Trichodolichopeza* and an identification key to Asian species is presented.

## Introduction

*Trichodolichopeza* Alexander, 1917 was established as a subgenus of the genus *Dolichopeza* Curtis, 1825 (Alexander 1917). It is a small subgenus, characterized by the following features: rostrum short, without a nasus; wing lacking Sc_1_; Sc_2_ entering R_1_ near the fork of Rs; Rs short; R_1+2_ short spur-like or entirely absent; discal cell absent; M_3_ originating prior to the fork of M_1+2_; and distal wing cells bearing microtrichia (Alexander 1917, 1964). The subgenus is predominantly distributed in the Afrotropical Region, with 24 known species. One species, D. (T.) sparsihirta Alexander, 1943, is known to occur in China (Alexander 1943; Oosterbroek 2025); this is the sole Dolichopeza (Trichodolichopeza) species in the Oriental Region. Knowledge of the immature stages and the biology of D. (Trichodolichopeza) remains limited, as only the immature stages of D. (T.) hirtipennis Alexander, 1917, D. (T.) peringueyi Alexander, 1925, and D. (T.) barnardi Wood, 1952 have been described (Wood 1952; Harrison and Barnard 1972). In the present study, two new species of D. (Trichodolichopeza) are described from China. Additionally, *D. fabella* Alexander, 1937, which was previously assigned to the subgenus D. (Nesopeza Alexander, 1914), is transferred to D. (Trichodolichopeza), based on the following characters: all species of the subgenus D. (Nesopeza) have bare cells, whereas this species possesses microtrichiae in the distal cells r_3_ to m_2_ and M_3_ (originated from M_1+2_); these are characters that are fully consistent with those of species in the subgenus D. (Trichodolichopeza). A taxonomic key to Asian species of D. (Trichodolichopeza) is provided.

## Materials and methods

Specimens were examined and illustrated using a ZEISS Stemi 2000-c stereomicroscope. Genitalic preparations were made by macerating the apical portion of the abdomen in cold 10% NaOH for 12–15 h. Following examination, preparations were transferred to fresh glycerine and stored in microvials pinned beneath the corresponding specimens. Type specimens examined are deposited in the Entomological Museum of China Agricultural University (**CAU**), Beijing. Other comparative material used in the current work include specimens from United States National Museum of Natural History, Washington, D.C., USA (**USNM**).

Morphological terminology follows Cumming and Wood (2017) for most characters. Terminology of male terminalia adheres to Ribeiro (2006). Wing cells are named after the vein lying above them. The following abbreviations are used in the figures: 9t, tergite 9; 9s, sternite 9; gx, gonocoxite; lg, lobe of gonostylus; cg, clasper of gonostylus; bbk, basal beak; bk, beak; dct, dorsal crest; pct, posterior crest.

## Taxonomy

### Key to Asian species of subgenus Dolichopeza (Trichodolichopeza)

**Table:** 

1	Thoracic pleura striped or lined with brown	**2**
–	Thoracic pleura uniform in colour, without stripes or lines	**3**
2	Cell m_2_ bare; abdominal tergites laterally with yellowish-white spots; posterior margin of tergite 9 depressed; sternite 9 prolonged, nearly 3 times as long as tergite 9	**D. (T.) caudata sp. nov**.
–	Cell m_2_ with microtrichiae; abdominal tergites with light yellowish-grey ring at midlength; male unknown	**Dolichopeza (Trichodolichopeza) sparsihirta Alexander, 1943**
3	Thoracic pleura dark brown; male unknown	**D. (T.) fabella Alexander, 1937 comb. nov**.
–	Thoracic pleura light yellow; tergite 9 with posterior margin produced medially, tip with a blunt protuberance; sternite 9 with lower tip produced into a rod, bent forwards	**D. (T.) uncinata sp. nov**.

#### Dolichopeza (Trichodolichopeza) caudata sp. nov.

Taxon classificationAnimaliaDipteraTipulidae

59465498-F657-5468-9B08-E051F4827C24

https://zoobank.org/7F770D29-3445-4616-B9D4-00FA1E7C2356

Figs 1–9

##### Type material.

***Holotype***: China • ♂ (pinned); Hainan Province; Baisha Li Autonomous County, Yuanmen Township, Hongmao Village; 19.083°N, 109.533°E, alt. 400 m; 21 May 2007 (1); Kuiyan Zhang leg.; net; CAU. ***Paratype***: China • ♀ (pinned); Hainan Province; Mount Wuzhishan; 18.867°N, 109.683°E, alt. 700 m; 28 May 2007 (1), Kuiyan Zhang leg.; net; CAU.

##### Diagnosis.

Antennal scape and pedicel yellowish brown. Wing greyish yellow; tips of distal cells brown; cell m_1_ half as long as its stalk; sparse microtrichiae present at extreme outer end of cell r_4+5_ to m_1_. Posterior margin of tergite 9 with a small, sharp, medial bulge and two small, short lateral lobes. Sternite 9 greatly prolonged. Lobe of gonostylus rod-like; beak of clasper of gonostylus pointed, with a deep notch on upper 1/3 of cephalad margin and a broad lobe medially produced.

##### Description.

**Male**. Body length 7.5 mm, wing length 8.0 mm.

**Figures 1–9.**
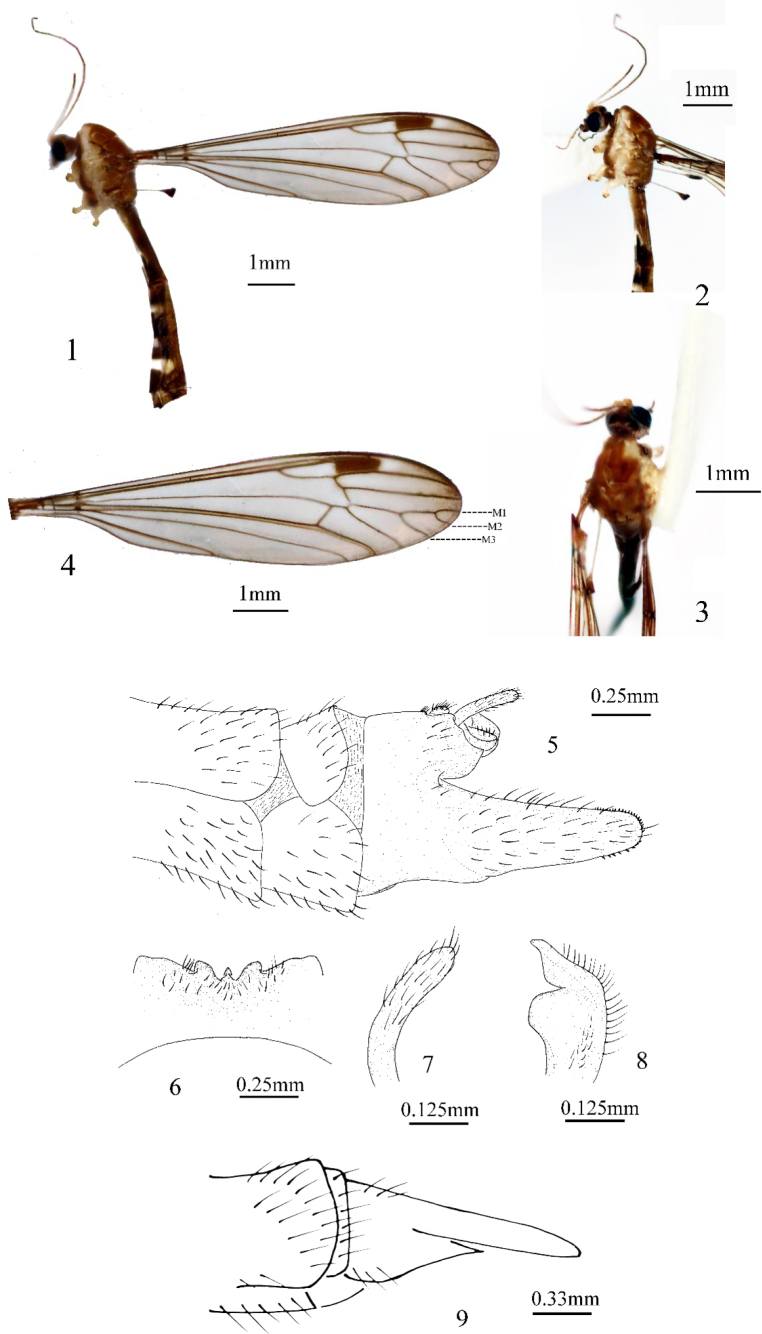
Dolichopeza (Trichodolichopeza) caudata sp. nov. **1–8**. Male: **1**. Body, lateral view; **2**. Head and thorax, lateral view; **3**. Head and thorax, dorsal view; **4**. Wing; **5**. Hypopygium, lateral view; **6**. Tergite 9, dorsal view; **7**. Lobe of gonostylus, lateral view; **8**. Clasper of gonostylus, lateral view; **9**. Female, ovipositor, lateral view.

***Head*** (Figs 1–3). Rostrum light brown, without a nasus. Vertex yellowish; occiput yellowish brown. Orbit yellowish. Antennal scape and pedicel yellowish brown; flagellum brown, with basal part of first flagellomere light brown. Labellum dark brown; palpus brownish.

***Thorax*** (Figs 1–3). General colouration yellowish brown. Pronotum yellowish brown; prescutum and presutural scutum yellowish, with yellowish-white cephalad lateral margins; scutum yellowish brown; scutellum yellowish brown; mediotergite yellowish brown, extending from neck to epimeron. Coxae and trochanters yellowish white, with front coxae basally yellowish brown; femora yellowish brown; tibiae dark yellowish brown; tarsi white, basally slightly yellowish brown. Setae on legs brown. Wing (Fig. 4) greyish yellow, with brown tips of distal cells; stigma brown; veins yellowish brown. Cell m_1_ half as long as its stalk. Sparse microtrichiae at extreme outer end of cells r_4+5_ to m_1_. Halter with a yellowish-brown stem and brown knob.

***Abdomen*** (Fig. 1). Ground colour yellowish brown. Tergites yellowish brown, with yellowish-white spots on the lateral margins of tergites 2–6. Sternites brownish black, medially with yellowish-white rings on sternites 2–6.

***Hypopygium*** (Figs 5–8). Tergite 9 and sternite 9 fused basally (Fig. 5). Tergite 9 (Fig. 6) brown, with posterior margin depressed, with a small, sharp, medial bulge and two small, short, lateral lobes. Sternite 9 prolonged, nearly 3 times as long as tergite 9 (Fig. 5). Lobe of gonostylus (Fig. 7) long and slender, rod-like, bearing long setae; clasper of gonostylus (Fig. 8) broad, beak-like, with a deep notch on upper 1/3 of cephalad margin, and a broad, medially produced lobe.

**Female**. Similar to male. Body length 9.0 mm, wing length 8.0 mm. Ovipositor (Fig. 9) obscurely yellowish brown. Cercus elongated, about 3 times as long as hypogynial valve, ventrally distinctly expanded at base; hypogynial valve strong, with pointed tip.

##### Etymology.

The specific epithet, *caudata* (Latin, “tailed”) is in reference to the prolonged sternite 9.

##### Distribution.

China (Hainan Province).

##### Remarks.

This new species is similar to D. (T.) sparsihirta in having a stripe on the thoracic pleura, but it can be easily distinguished by the following features: cell m_2_ lacking microtrichiae, and abdominal tergites 2–6 with yellowish-white spots on lateral margins. In D. (T.) sparsihirta, cell m_2_ possesses microtrichiae, and the abdominal tergites have a narrow, light yellowish-grey ring near midlength (Alexander 1943).

#### Dolichopeza (Trichodolichopeza) uncinata sp. nov.

Taxon classificationAnimaliaDipteraTipulidae

71292515-3ACB-519D-A895-B88324ED9ABF

https://zoobank.org/47B235F5-199E-4FF9-98B4-47470ABB785B

Figs 10–17

##### Type material.

***Holotype***: China • ♂ (pinned); Henan Province; Huixian City, Baligou Scenic Area; 35.583°N, 113.583°E, alt. 331 m; 13 July 2007 (1); Caixia Gao leg.; net; CAU.

##### Diagnosis.

Antennal scape and pedicel yellow, flagellum dark yellowish brown. Prescutum and presutural scutum yellowish, with three medial golden-yellow stripes. Thoracic pleura yellowish. Wing light greyish yellow, with cell m_1_ as long as its stalk, with sparse microtrichiae present at the extreme outer end of cells r_3_ to r_4+5_. Tergite 9 black; posterior margin medially produced, with a blunt protuberance on lower margin and two small, sharp, lateral points. Sternite 9 with lower tip produced into a forward-bent rod. Lobe of gonostylus long, slender, rod-like; lower posterior margin of clasper of gonostylus with a broad lobe.

##### Description.

**Male**. Body length 8.0 mm, wing length 9.0 mm.

***Head*** (Figs 11–13) light yellow. Rostrum short, light yellow, without a nasus. Vertex yellowish; occiput dark yellow. Orbit yellowish. Antennal scape and pedicel yellow; flagellum dark yellowish brown. Labellum brown; palpus reddish brown.

**Figures 10–17.**
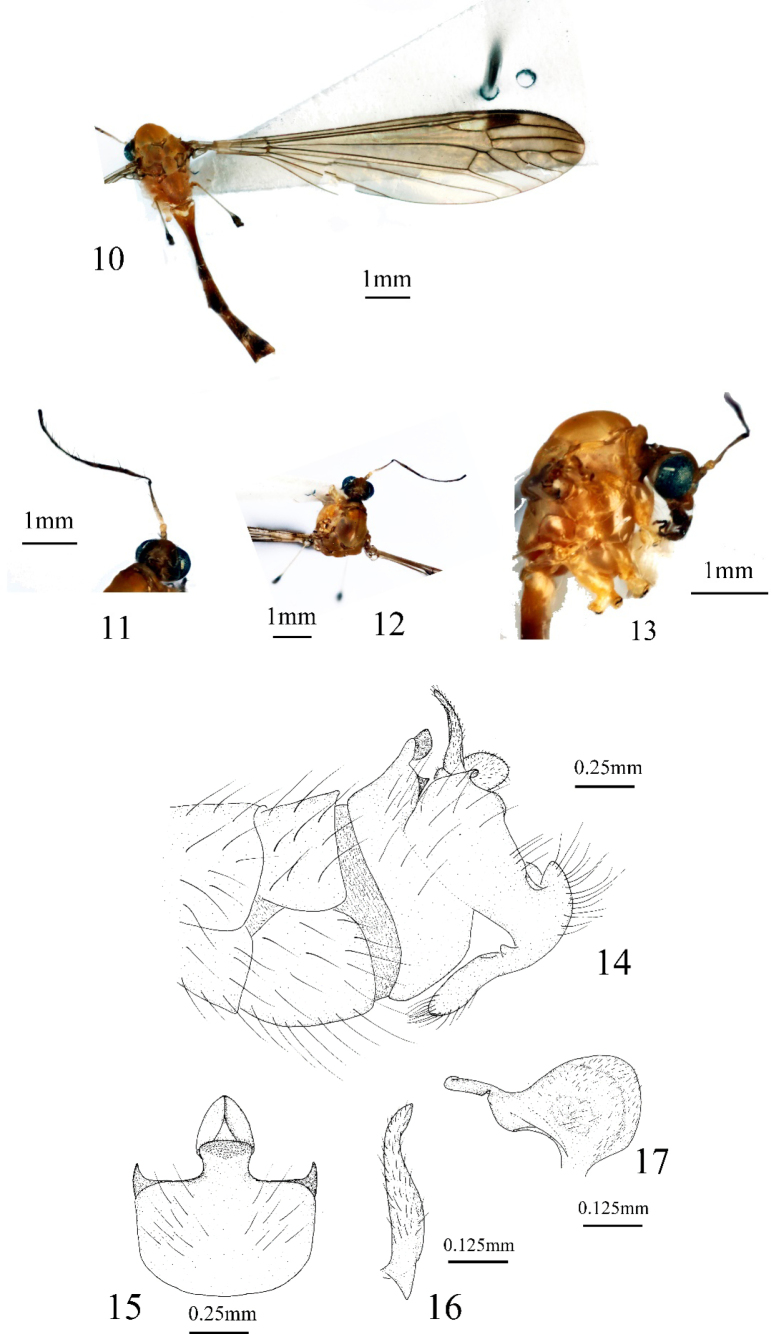
Dolichopeza (Trichodolichopeza) uncinata sp. nov. **10**. Body, dorsal view; **11**. Head, dorsal view; **12**. Head and thorax, dorsal view; **13**. Head and thorax, lateral view; **14**. Hypopygium, lateral view; **15**. Tergite 9, dorsal view; **16**. Lobe of gonostylus, lateral view; **17**. Clasper of gonostylus, lateral view.

***Thorax*** (Figs 10, 12, 13). General colouration yellowish. Pronotum yellowish; prescutum and presutural scutum yellowish, with three golden-yellow medial stripes; scutum golden yellow, with yellowish lateral margins; scutellum yellowish; mediotergite golden yellow. Pleura yellowish, unpatterned. Coxae and trochanters yellowish; other leg segments broken. Wing (Fig. 10) light greyish yellow; stigma light brown; veins yellowish brown. Cell m_1_ as long as its stalk. Sparse microtrichiae at extreme outer end of cells r_3_ to r_4+5_. Halter with a yellowish-white stem and a dark-brown knob.

***Abdomen*** (Fig. 10). Ground colour yellowish brown. Tergites yellowish brown, basally with narrow, dark-yellow rings.

##### Sternites yellowish brown.

***Hypopygium*** (Figs 14–17). Tergite 9 and sternite 9 basally fused. Tergite 9 (Fig. 15) black, with posterior margin slightly sclerotized, produced medially into a blunt protuberance on lower margin and two small, sharp lateral points. Sternite 9 with lower tip produced into a forward-bent rod. Lobe of gonostylus (Fig. 16) long, slender, rod-like, apically narrowed, bearing short setae. Clasper of gonostylus (Fig. 17) basally bent forward; with blunt beak, with a small notch at upper 1/3 of the cephalad margin, slightly produced medially, and with a broad lobe produced on the lower posterior margin.

**Female**. Unknown.

##### Distribution.

Only known from the type locality.

##### Remarks.

This new species is similar to *D. fabella* in having unpatterned thoracic pleura, but it can be easily distinguished by the yellowish colour of the thoracic pleura. In *D. fabella*, the thoracic pleura is dark brown (Alexander 1937).

##### Etymology.

The specific epithet, *uncinata* (Latin, “hooked”) is in reference to the forward-bent (hooked) projection of sternite 9.

#### Dolichopeza (Trichodolichopeza) fabella

Taxon classificationAnimaliaDipteraTipulidae

Alexander, 1937 comb. nov.

B33EB624-FBA5-55E1-BFFC-E3DFC0CE3130

Fig. 18

Dolichopeza (Nesopeza) fabella Alexander, 1937: 370. Type locality: China: Guangdong Province: Taiyong (presumed to be Humen, Dongguan City, Guangdong Province).

##### Specimens examined.

China • Holotype, gender unknown (pinned); Guangdong Province, Guangzhou city, Tai-yong; alt. 2075 feet; 6 August 1936 (1); L. Gressitt leg.; USNM.

**Figure 18.**
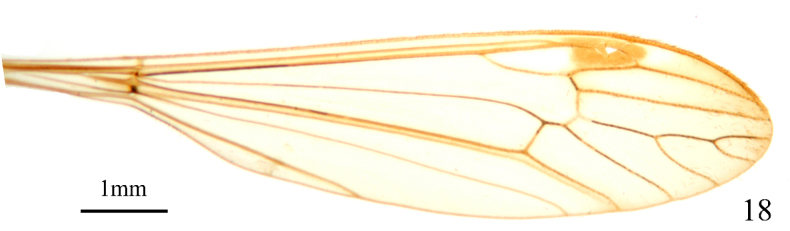
Wing slide of Dolichopeza (Trichodolichopeza) fabella Alexander, 1937, holotype.

##### Diagnosis.

Antennal scape brown; pedicel yellowish brown; flagellum with first segment yellowish brown and other segments blackish brown. Mesonotal prescutum brown, with a faint, dark-brown medial stripe. Thoracic pleura dark brown. Wing yellow, with tip yellowish brown, cell m_1_ as long as its stalk, sparse microtrichiae present at extreme outer end of cells r_3_ to m_2_.

##### Description.

Abdomen incomplete, gender unknown. Wing length 9.0 mm.

***Head***. Brown. Rostrum short, brown, without a nasus. Vertex brown; occiput brown. Antennal scape brown; pedicel yellowish brown; first segment of flagellum yellowish brown and other segments blackish brown. Labellum dark brown; palpus blackish brown.

***Thorax***. Brown. Pronotum dark brown; mesonotal prescutum brown, with yellow cephalad lateral margins and a faint, dark-brown medial stripe; scutellum brown; mediotergite dark brown. Pleura dark brown, unpatterned. Front coxae and trochanters dark brown; middle and hind coxae and trochanters brown. Only hind legs preserved; femora yellowish brown, with extreme tips brown; tibiae dark brown; tarsi dark brown. Wing (Fig. 18) yellow, with a light yellowish-brown tip; stigma yellowish brown, with white spots on both sides; veins yellowish brown; wing with long hairs on veins beyond the cord. Cell m_1_ as long as its stalk. Sparse microtrichiae at extreme outer end of cells r_3_ to m_2_. Cell a_2_ narrow. Halter with a yellowish stem; knob brown.

***Abdomen***. Apically broken. Tergites dark brown, with white spots medially on lateral margins. Sternites blackish brown, with a white, medial ring on second sternite.

##### Distribution.

China (Guangdong Province).

##### Remarks.

This species was originally assigned to the subgenus D. (Nesopeza) by Alexander (1937); however, *Nesopeza* species all have the cells bare. In contrast, this species processes microtrichiae in the distal cells r_3_ to m_2_ and M_3_ (which originates from M_1+2_). These characters are consistent with *Trichodolichopeza* species. Therefore, it is transferred to the subgenus D. (Trichodolichopeza). This transfer was suggested in the unpublished thesis of one of the authors (Liu 2011), but the transfer is formally proposed and effectively published here for the first time.

## Supplementary Material

XML Treatment for Dolichopeza (Trichodolichopeza) caudata

XML Treatment for Dolichopeza (Trichodolichopeza) uncinata

XML Treatment for Dolichopeza (Trichodolichopeza) fabella

## Additional information

### Artificial Intelligence (AI) use

The authors accept full responsibility for the content of the manuscript, including the disclosure of any use of AI.

No AI tools were used in the preparation of this manuscript.

### Data availability

All of the data that support the findings of this study are available in the main text.
